# Safety, effectiveness and healthcare resource implications of an early discharge pathway for cryoballoon ablation: real-world evidence from China

**DOI:** 10.3389/fcvm.2026.1969824

**Published:** 2026-10-26

**Authors:** Huashan Huang, Binbin Chen, Xuewen Liao, BaoJian Zuo, Lianghua Lian, Changsheng Fan, Zhiping Yang, Yazhou Lin

**Affiliations:** 1 Department of Cardiology, Fuzhou University Affiliated Provincial Hospital, Fujian Provincial Hospital, Fuzhou, China; 2 Shengli Clinical Medical College of Fujian Medical University, Fuzhou, China; 3 Beijing Medical and Health Economic Research Association, Beijing, China

**Keywords:** cryoballoon ablation, early discharge pathway, healthcare resource implications, propensity score matching, real-world study

## Abstract

**Introduction:**

Same-day discharge after atrial fibrillation (AF) ablation has been increasingly adopted in Western healthcare systems. However, its applicability in China remains uncertain because of differences in healthcare delivery, peri-procedural management, and hospitalization practices. Meanwhile, real-world evidence on early discharge pathways for AF ablation in the Chinese setting remains limited. This study evaluated the clinical and economic outcomes of an early discharge pathway for patients undergoing cryoballoon ablation in China.

**Methods:**

A retrospective real-world study was conducted using data from the institutional atrial fibrillation database at Fuzhou University Affiliated Provincial Hospital. Consecutive patients who underwent cryoballoon ablation between January 2021 and December 2023 were included. Patients were stratified into two cohorts based on post-operative length of stay: the early discharge (ED) group (discharged within 48 h) and the conventional inpatient (CI) group (discharged after 48 h). Propensity score matching was performed to minimize baseline imbalances between groups. The outcomes were atrial fibrillation recurrence, post-procedural complications and hospitalization cost.

**Results:**

A total of 327 patients were included in the final analysis, with 151 in the ED group and 176 in the CI group. After 1:1 propensity score matching, 80 patients remained in each group, and baseline comparability was substantially improved after matching, although a small residual imbalance remained for stroke/TIA history. During 12 months of follow-up, the recurrence rate of AF was similar between the two groups (8.75% vs. 10.00%, *P* = 0.786). No major in-hospital complications occurred in the ED group, whereas one case of pericardial effusion was reported in the CI group. Subgroup analyses showed no significant difference in recurrence among patients aged 65 years or older or among those with persistent atrial fibrillation. The ED group incurred significantly lower medical service costs (*P* = 0.0003) and medication costs (*P* = 0.035) than the CI group. However, no significant difference was observed in total hospitalization costs between the two groups (*P* = 0.957).

**Discussion:**

In this real-world Chinese cohort, a structured early discharge pathway for cryoballoon ablation appeared to be feasible and was not associated with a higher AF recurrence rate, while reducing medical service-related costs. However, because this was a retrospective single-center study with a limited sample size and no systematic post-discharge surveillance for all potential events, these findings should be interpreted with caution. Larger prospective studies are needed to further confirm the safety, effectiveness, and economic implications of this model.

## Introduction

Atrial fibrillation (AF) is the most common sustained cardiac arrhythmia and remains a major contributor to morbidity, mortality, and healthcare utilization worldwide. Despite advances in prevention and treatment, the burden of AF continues to increase owing to an aging population and improved survival of patients with cardiovascular disease (1). AF is associated with a markedly increased risk of stroke, heart failure, cognitive impairment, and death, creating substantial challenges for healthcare systems (2, 3). In China, more than 4.8 million adults aged ≥35 years are estimated to be living with AF (4). Catheter ablation has become a well-established treatment strategy for patients with symptomatic AF and is recommended by contemporary guidelines as a first-line treatment option in selected patients with symptomatic AF (5, 6). As the prevalence of AF continues to rise and catheter ablation continues to expand, optimizing post-procedural care and hospitalization strategies has become increasingly important.

Given the increasing demand for AF ablation, same-day discharge protocols have been widely adopted to mitigate healthcare resource constraints (7). Growing evidence from Europe and North America supports the feasibility and safety of this approach in appropriately selected patients. A systematic review and meta-analysis of more than 18,000 AF ablation procedures reported a successful same-day discharge (SDD) rate of approximately 90%, with low rates of major complications and early readmissions or emergency department visits (8). Moreover, an observational study has shown that SDD is feasible after both radiofrequency and cryoablation, with low rates of post-discharge complications and no apparent differences in readmission or emergency department visits between ablation modalities (9). Supporting these observational findings, the EASY PVI study, the first randomized trial evaluating SDD after cryoablation, reported no significant difference in short-term adverse events between SDD and overnight hospitalization among carefully selected patients with paroxysmal AF (10).

Although same-day discharge has become a well-established approach in many Western healthcare systems, substantial differences remain between international and Chinese practice. In the United States and the United Kingdom, more than 80% of elective surgical cases are managed through same-day discharge pathways, reflecting the maturity of ambulatory surgical systems and standardized discharge processes (11). In contrast, day surgery procedures accounted for less than 20% of elective procedures in Chinese tertiary public hospitals in 2023, indicating considerable room for further development (12, 13). Distinctively, the Chinese day surgery model can be understood as a localized adaptation of an early discharge pathway, typically involving structured post-procedural observation and discharge within 48 h in a hospital-based setting (14). Against this background, whether evidence supporting same-day discharge after AF ablation in Western healthcare systems can be directly generalized to Chinese clinical practice remains uncertain. This concern is further underscored by differences in healthcare delivery systems, peri-procedural management pathways, discharge practices, and resource organization, which may influence both clinical and economic outcomes (14).

Meanwhile, the increasing prevalence of AF and the growing demand for catheter ablation continue to place substantial pressure on inpatient bed capacity and healthcare resources in high-volume tertiary centers across China. Under these circumstances, structured early discharge pathways may offer an opportunity to improve healthcare efficiency while maintaining procedural safety. However, despite the rapid expansion of early discharge services in China, high-quality evidence supporting many current recommendations remains limited (15), and procedure-specific data for AF ablation are particularly scarce (16, 17). Therefore, we conducted a real-world study at a high-volume cryoballoon ablation center in China to evaluate the clinical outcomes and healthcare resource implications of a structured early discharge pathway compared with conventional inpatient hospitalization.

## Methods

### Study design and data sources

This study was a single-center, retrospective observational cohort study conducted at Fuzhou University Affiliated Provincial Hospital, China. Data were extracted from a dedicated atrial fibrillation registry database that systematically collects information from the electronic medical record system (EMR) and hospital information system (HIS). The study protocol was reviewed and approved by the hospital ethics committee (approval number: K2026-03-013), and patient informed consent was waived for retrospective anonymous data analysis in accordance with institutional guidelines. Consecutive patients who underwent cryoballoon ablation for atrial fibrillation (AF) between January 2021 and December 2023 were included. According to the length of hospital stay, patients were categorized into either the early discharge (ED) group or the conventional inpatient (CI) group. Patients in the ED group were discharged within 48 h after the procedure, whereas those in the CI group followed the standard inpatient care pathway and were discharged more than 48 h. In addition to the overall cohort analyses, prespecified subgroup analyses were performed according to age and AF type (paroxysmal AF and persistent AF).

### Study population

The study fulfilled the following criteria: (1) patients with age ≥ 18, with paroxysmal or persistent AF undergoing cryoballoon ablation for the first time; (2）procedures conducted from January 2021 to December 2023. The patient selection process is illustrated in Figure 1.

**Figure 1:**
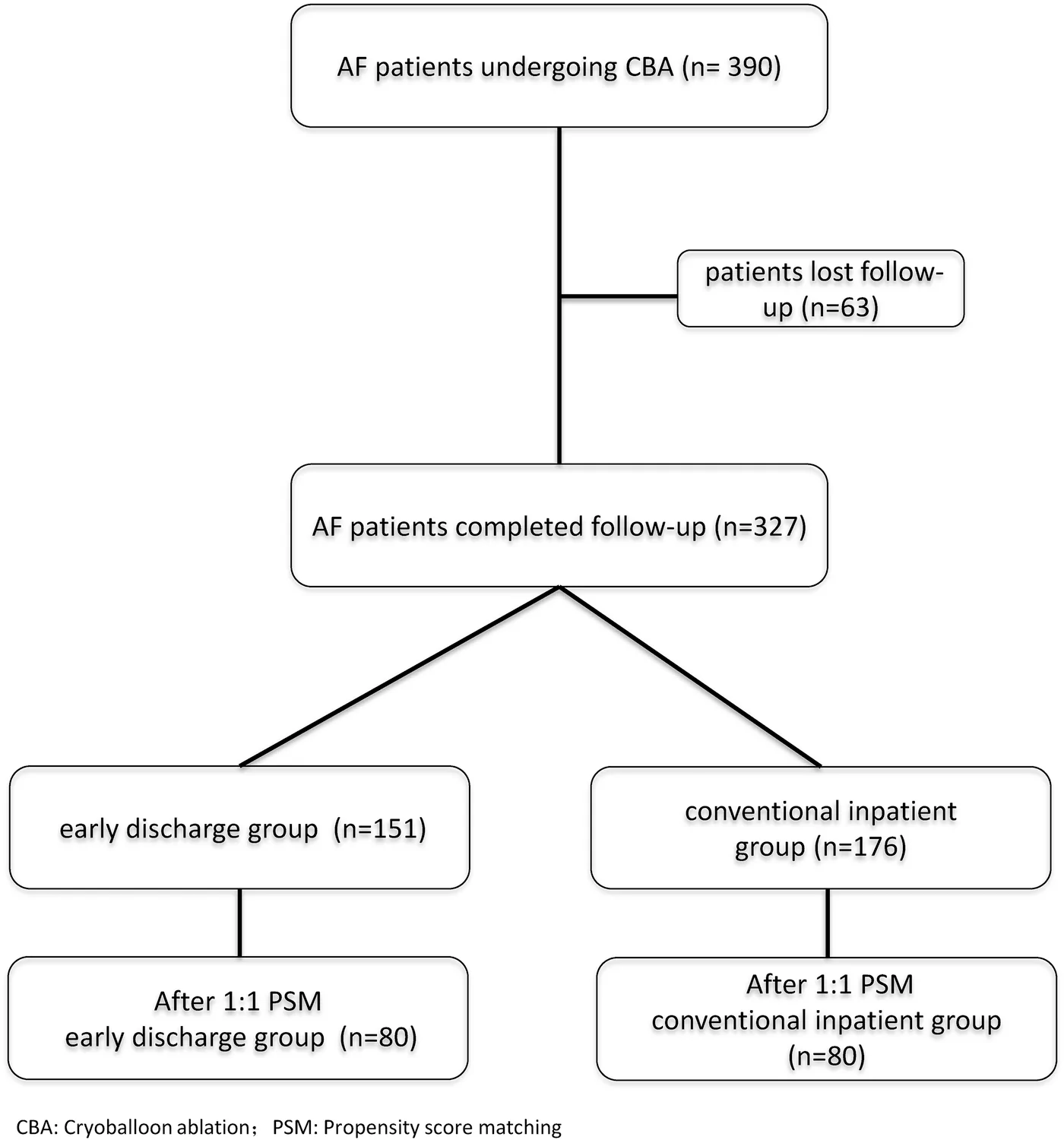
Flow diagram of patient selection.

### Ablation procedure and pre-discharge assessment

All procedures were performed under local anesthesia with conscious sedation using intravenous fentanyl and midazolam. Left common femoral vein cannulation was performed twice sequentially using the Seldinger technique. A quadripolar catheter and a deflectable decapolar catheter (6F, Abbott, US) were positioned in the right ventricle and coronary sinus, respectively. Following right common femoral vein cannulation, a single fluoroscopy-guided transseptal puncture was performed, and intravenous heparin was given to maintain intraoperative ACT at 300–350 s. Cryoballoon ablation was performed using the Arctic Front system and FlexCath steerable sheath (Medtronic, Minneapolis, MN). A 28-mm balloon and proximal-seal technique were used for all pulmonary veins. All sheaths were removed upon procedure completion. A figure-of-eight suture was placed at the right common femoral vein access site for hemostasis, while manual compression was applied to the left common femoral vein puncture site. Typically, three venous access sites were obtained from the bilateral femoral veins for all procedures, with two punctures on the left side and one on the right. The same vascular closure protocol was applied uniformly to all patients in both groups.

Patients were transferred to the inpatient ward for routine post-procedural monitoring. Continuous ECG monitoring was maintained for at least 6 h, and vital signs along with vascular access sites were assessed hourly to detect any signs of bleeding, hematoma, or pseudoaneurysm. Routine laboratory testing was not performed unless clinically indicated. Transthoracic echocardiography (TTE) was not routinely performed before discharge; it was reserved for patients exhibiting clinical signs suggestive of pericardial effusion, such as unexplained hypotension, tachycardia, or chest pain.

Patients were allowed to ambulate 6 h post-procedure and the sutures were removed before hospital discharge. Complete hemostasis was defined as the absence of pain, active bleeding, or expanding hematoma at the access sites after ambulation. Patients were eligible for discharge within 48 h only if they met the following specific criteria: (1) stable vital signs for at least 6 h prior to discharge; (2) sinus rhythm or rate-controlled atrial arrhythmia without hemodynamic compromise; (3) no evidence of vascular complications at the access sites; (4) ability to ambulate independently without dizziness or syncope; and (5) the presence of a responsible adult to accompany the patient home.

Patients undergoing AF ablation who were considered suitable for early discharge were managed through a structured early discharge pathway, in accordance with institutional practice. In addition to these discharge criteria, actual discharge timing in routine clinical practice could also be influenced by non-clinical factors, including patient preference, availability of family support at home, and logistical considerations related to hospital workflow and weekend discharge arrangements.

### Outcomes

The baseline characteristics included: age, sex, BMI, atrial fibrillation types, CHA_2_DS_2_-VASc score, left ventricular ejection fraction (LVEF), concomitant left atrial appendage occlusion (LAAO) and comorbidities. The clinical outcomes of the study were the postoperative recurrence rate of AF and the complications. AF recurrence was defined as any ECG-documented AF episode occurring beyond the 3-month post-procedure blanking period. Patients were routinely followed at 3, 6, and 12 months after the procedure. Standard 12-lead ECG examinations were routinely performed at 3, 6, and 12 months after the procedure. No routine Holter monitoring, implantable loop recorders, wearable monitoring devices, or other standardized continuous rhythm-monitoring strategies were used during follow-up. Between visits, monitoring was symptom-driven, and asymptomatic episodes were not systematically captured. Procedure-related adverse events were recorded only through hospital discharge; post-discharge complications, emergency department visits, and readmissions were not systematically assessed. The economics outcomes included: total hospitalization cost, diagnostic costs, medical services costs, drug costs, consumables costs.

### Statistical analysis

Continuous variables were summarized as mean with standard deviation (SD), median with interquartile range (IQR), and minimum and maximum values, whereas categorical variables were presented as frequencies and percentages. For comparisons between the early discharge (ED) and conventional inpatient (CI) groups, continuous variables were compared using the Kruskal–Wallis test, and categorical variables were compared using the chi-square test or Fisher's exact test, as appropriate.

To reduce confounding bias caused by differences in baseline characteristics, propensity score matching (PSM) was performed. Propensity scores were estimated using a multivariable logistic regression model, with discharge pathway (ED vs CI) as the dependent variable and baseline covariates as independent variables. Matching variables included age, gender, body mass index, type of atrial fibrillation (paroxysmal and persistent), CHA2DS2-VASc scores, left ventricular ejection fraction (LVEF), left atrial appendage occlusion (LAAO), coronary artery disease, stroke/TIA history, hypertension, diabetes, hyperlipidaemia, kidney disease, and liver disease**.** Patients in the ED and CI groups were matched at a 1:1 ratio using nearest-neighbour matching with a caliper width of 0.1. Covariate balance before and after matching was assessed using standardized mean differences (SMDs). For continuous variables, SMDs were calculated as the between-group difference in means divided by the pooled SD. For categorical variables, SMDs were calculated based on differences in proportions; for multi-category variables, the maximum absolute category-specific SMD was reported. Given that no single cutoff is universally accepted for all covariates in real-world PSM analyses, balance was assessed using standardized mean differences and visual inspection of a Love plot.

Time-to-event data for AF recurrence were analysed using Kaplan–Meier survival analysis, and between-group comparisons were performed using the log-rank test. In the propensity score-matched cohort, Kaplan–Meier curves were generated for the overall population and for prespecified subgroup analyses stratified by age (<65 vs. ≥ 65 years) and AF type (persistent vs. paroxysmal AF). Because this was an exploratory real-world study with multiple clinical and economic outcomes, *P* values from subgroup analyses were interpreted descriptively. All statistical analyses were performed using SAS version 9.4. A two-sided alpha level of 0.05 was used, and *P* < 0.05 was considered statistically significant.

## Results

### Baseline characteristics

A total of 390 consecutive patients were diagnosed with AF and underwent cryoballoon ablation from January 2021 to December 2023 in the hospital. 63 patients were excluded due to missing follow-up data. Among the 327 included patients, 151 were managed through an early disharge pathway and 176 received conventional inpatient care. Following 1:1 propensity score matching, baseline comparability between the two groups was substantially improved. Most demographic variables and comorbidities, including age, LVEF, AF type, CHA2DS2-VASc score, left atrial appendage occlusion (LAAO), coronary artery disease, diabetes, hyperlipidaemia, kidney disease, liver disease, were well balanced between groups. Standardized mean differences were below 0.1 for most covariates, indicating substantial improvement in baseline comparability after matching. Mild residual imbalance remained for gender, BMI, hypertension and stroke/ TIA history, with SMDs of 0.160, 0.115, 0.126 and 0.219, respectively. After propensity score matching, baseline comparability between the two groups was substantially improved, with only minor residual imbalance remaining, as demonstrated by standardized mean differences and visual assessment using the Love plot. Details are presented in Table 1 and Figure 2.

**Table 1:** Baseline characteristics before and after propensity score matching.

Variables	Before PSM early discharge (*N* = 151)	Before PSM conventional inpatient (*N* = 176)		SMD	*P*-value	After PSM early discharge (*N* = 80)	After PSM conventional inpatient (*N* = 80)	SMD	*P*-value
Age				0.109	0.314^a^			0.029	0.739^a^
Total	151		176			80	80		
Mean (standard deviation)	63.77 (10.7)		64.93 (10.58)			63.13 (11.22)	63.43 (9.15)		
Median (IQR)	65 (60,71)		67 (59,72.5)			65 (57.5,71)	64.5 (58,69)		
Minimum, maximum	24,86		32,85			35,84	33,80		
Gender, *n* (%)				0.030	0.814^b^			0.161	0.311^c^
male	100 (66.23)		119 (67.61)			57 (71.25)	51 (63.75)		
female	51 (33.77)		57 (32.39)			23 (28.75)	29 (36.25)		
Body mass index (BMI)				0.071	0.312^a^			0.115	0.245^a^
Total (missing number)	115 (36)		142 (34)			80	80		
Mean (standard deviation)	25.02 (3.53)		24.77 (3.5)			25.25 (3.94)	24.83 (3.41)		
Median (IQR)	25.22 (22.89,26.63)		24.31 (22.66,26.7)			25.14 (22.96,26.96)	24.03 (22.82,26.6)		
Minimum, maximum	13.31,39.34		15.82,36.68			13.31,39.34	17.07,36.68		
AF type, *n* (%)				0.235	0.035^c^			0.026	1.000^b^
Paroxysmal atrial fibrillation	98 (64.90)		94 (53.41)			48 (60.00)	47 (58.75)		
Persistent atrial fibrillation	53 (35.10)		82 (46.59)			32 (40.00)	33 (41.25)		
CHA2DS2-VASc score				0.285	0.010^a^			0.063	0.834^a^
Total	151 (0)		176 (0)			80	80		
Mean (standard deviation)	1.98 (1.42)		2.4 (1.55)			1.99 (1.44)	1.9 (1.35)		
Median (IQR)	2 (1,3)		2 (1,3)			2 (1,3)	2 (1,3)		
Minimum, maximum	0,6		0,6			0,6	0,6		
Left ventricular ejection fraction (LVEF)				0.206	0.242^a^			0.026	0.496^a^
Total (missing number)	134 (17)		169 (7)			80	80		
Mean (standard deviation)	59.73 (5.2)		58.72 (4.62)			59.16 (5.03)	59.29 (4.61)		
Median (IQR)	60 (57,62)		59 (57,61)			59 (56.5,62)	60 (57,62)		
Minimum, maximum	43,79		40,70			43,75	40,70		
Left atrial appendage occlusion (LAAO), *n* (%)				0.601	<.0001^b^			0.000	1.000^c^
no	132 (87.42)		110 (62.50)			66 (82.50)	66 (82.50)		
yes	19 (12.58)		66 (37.50)			14 (17.50)	14 (17.50)		
Coronary artery disease, *n* (%)				0.100	0.423^b^			0.037	1.000^b^
no	133 (88.08)		149 (84.66)			70 (87.50)	69 (86.25)		
yes	18 (11.92)		27 (15.34)			10 (12.50)	11 (13.75)		
Stroke/TIA history, *n* (%)				0.072	0.566^b^			0.219	0.168^c^
no	126 (83.44)		142 (80.68)			66 (82.50)	72 (90.00)		
yes	25 (16.56)		34 (19.32)			14 (17.50)	8 (10.00)		
Hypertension, *n* (%)				0.037	0.737^c^			0.126	0.526^b^
no	83 (54.97)		100 (56.82)			45 (56.25)	40 (50.00)		
yes	68 (45.03)		76 (43.18)			35 (43.75)	40 (50.00)		
Diabetes, *n* (%)				0.062	0.677^b^			0.031	1.000^b^
no	123 (81.46)		139 (78.98)			63 (78.75)	64 (80.00)		
yes	28 (18.54)		37 (21.02)			17 (21.25)	16 (20.00)		
Hyperlipidemia, *n* (%)				0.003	1.000^b^			0.000	1.000^c^
no	116 (76.82)		135 (76.70)			58 (72.50)	58 (72.50)		
yes	35 (23.18)		41 (23.30)			22 (27.50)	22 (27.50)		
Kidney disease, *n* (%)				0.055	0.623^c^			0.000	1.000^b^
no	145 (96.03)		167 (94.89)			76 (95.00)	76 (95.00)		
yes	6 (3.97)		9 (5.11)			4 (5.00)	4 (5.00)		
Liver disease, *n* (%)				0.041	0.714^c^			0.049	0.755^c^
no	143(94.70)		165(93.75)			75(93.75)	74(92.50)		
yes	8(5.30)		11(6.25)			5(6.25)	6(7.50)		

a Stands for Kruskal–Wallis test.

b Stands for Fisher exact test.

c Stands for chi square test.

Q1: lower quartile; Q3: upper quartile. SMD: standardized mean difference. For multi-category variables, the variable-level SMD is the maximum absolute category-specific SMD; category rows do not display SMD.

**Figure 2:**
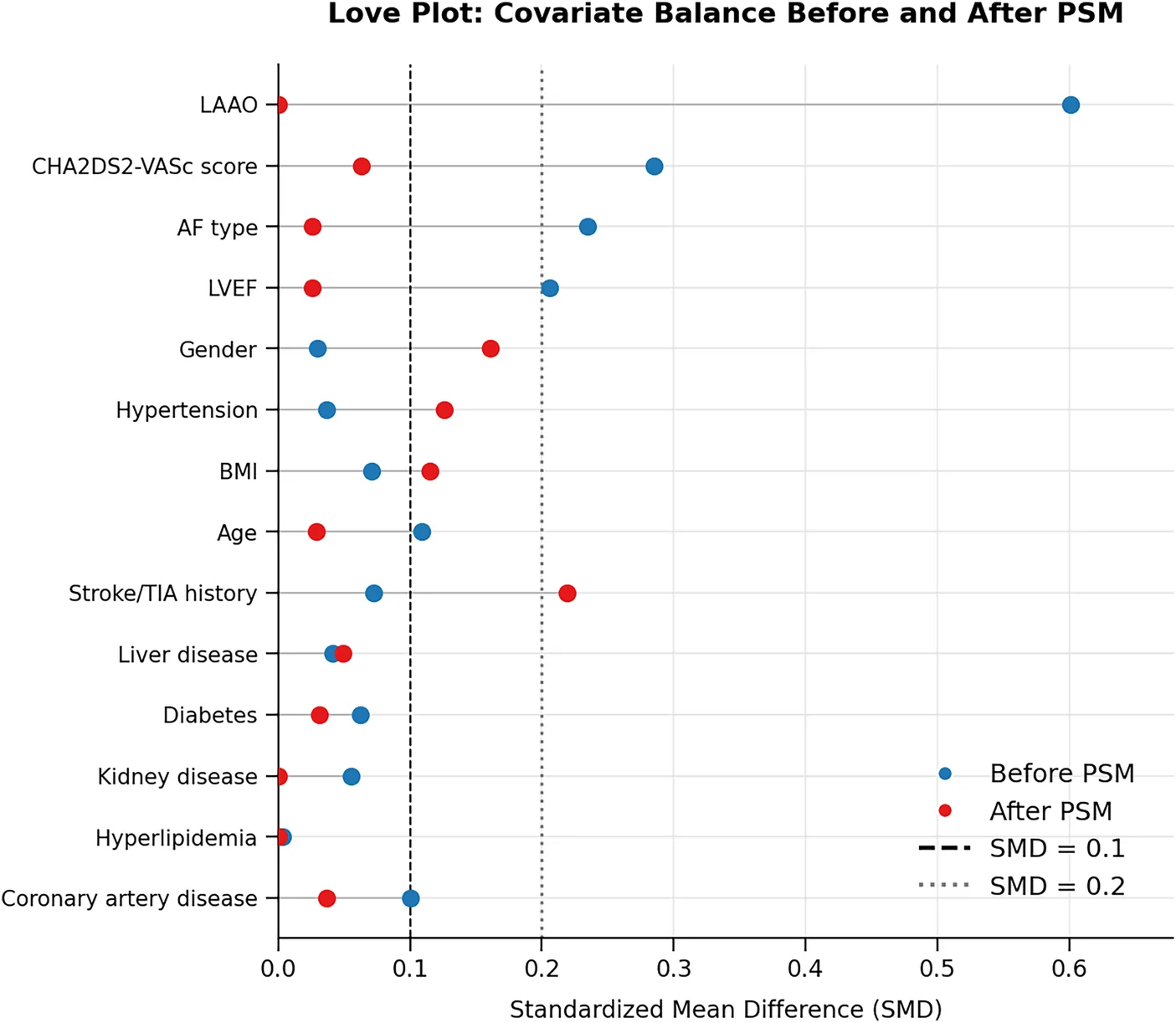
Love plot of covariate balance before and after PSM.

### Clinical outcome

No statistically significant differences in AF recurrence were observed between the two groups at 3 months, 6 months, or 12 months. At 3 months, the AF recurrence rate was 1.25% in both the ED and CI groups (*P* = 1.000). At 12 months, AF recurrence occurred in 8.75% of patients in the ED group and 10.00% of patients in the CI group (*P* = 0.786). Details are presented in Table 2. After propensity score matching, Kaplan–Meier analysis also showed no significant difference in AF recurrence within 12 months between the ED group and the CI group (log-rank *P* = 0.517). The median time to recurrence was not reached in either group. Details are presented in Figure 3.

**Table 2:** Recurrence outcomes during follow-up in the early discharge and inpatient groups.

Variable	ED group (*N* = 80)	CI group (*N* = 80)	Total (*N* = 160)	*P* value
Recurrence status at 3 months, *n* (%)				1.000^b^
No recurrence	79 (98.75)	79 (98.75)	158 (98.75)	
Recurrence	1 (1.25)	1 (1.25)	2 (1.25)	
Recurrence status at 6 months, *n* (%)				1.000^b^
No recurrence	77 (96.25)	78 (97.50)	155 (96.88)	
Recurrence	3 (3.75)	2 (2.50)	5 (3.13)	
Recurrence status at 12 months, *n* (%)				0.786^a^
No recurrence	73 (91.25)	72 (90.00)	145 (90.63)	
Recurrence	7 (8.75)	8 (10.00)	15(9.38)	

a Stands for chi-square test.

b Stands for Fisher test.

**Figure 3:**
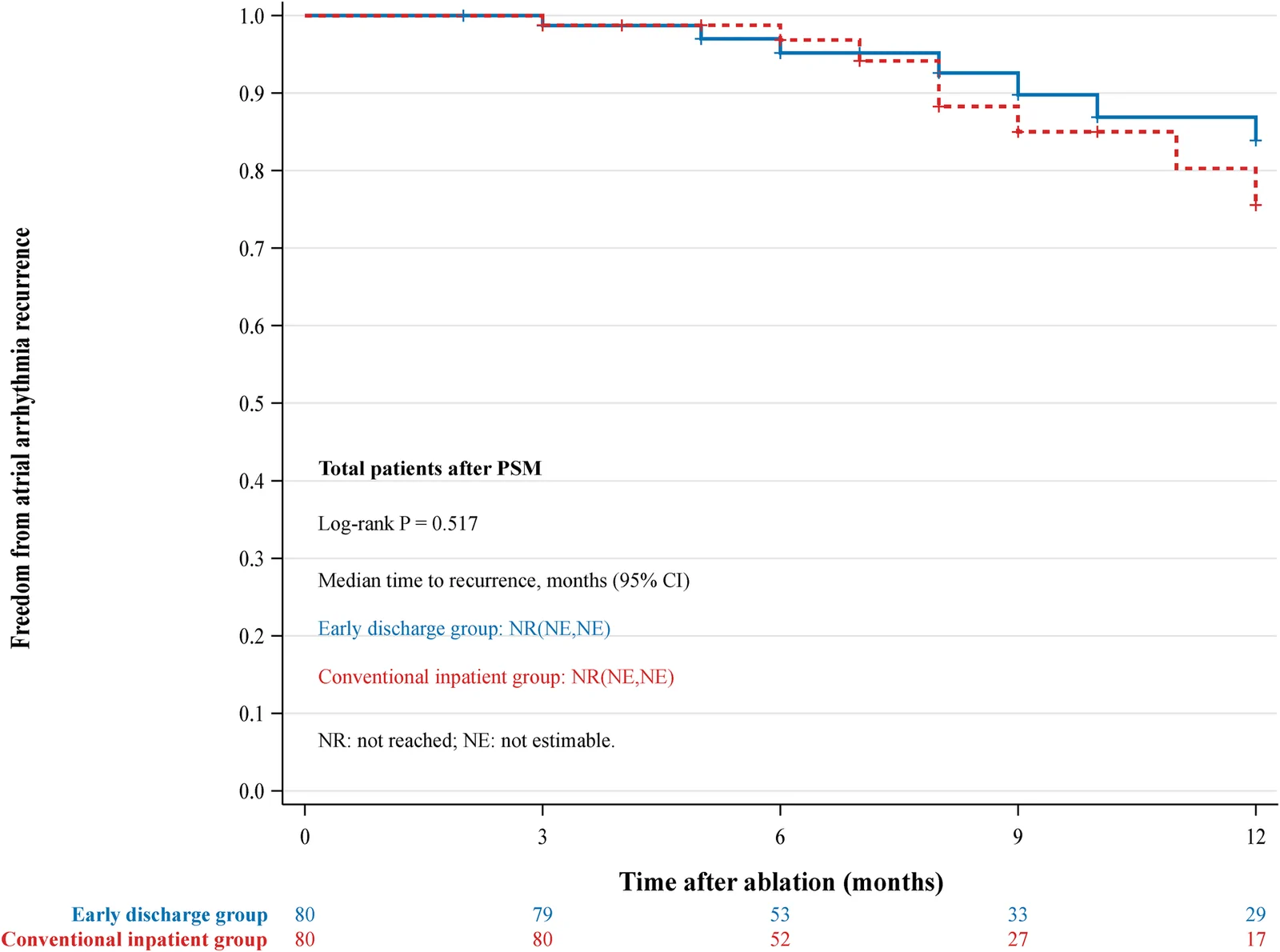
Kaplan–meier curves for AF recurrence in the two groups.

There was no significant difference in the number of complications between the ED and CI cohorts within 48 h following the cryoballoon ablation. Only one pericardial effusion occurred in the CI cohort, and it was managed promptly by the physician during the procedure. The ED cohort had no complication within 48 h. However, because post-discharge adverse events were not systematically assessed, late complications cannot be excluded.

The time from surgery to discharge differed significantly between the two groups (*P* < 0.0001). In the ED group, 42 patients (52.50%) were discharged within 12 to 24 h, 11 (13.75%) within 24 to 36 h, and 27 (33.75%) within 36 to 48 h. In contrast, no patients in the CI group were discharged within 48 h; 33 patients (41.25%) were discharged within 48 to 72 h, and 47 (58.75%) after more than 72 h. Furthermore, the length of hospital stay was significantly shorter in the ED group (*P* < 0.0001). Among ED patients, 53 (66.25%) were discharged after one night of hospitalization and 27 (33.75%) after two nights, whereas all patients in the CI group remained hospitalized for at least three nights before discharge. Details are presented in Table 3.

**Table 3:** Time distribution from surgery to discharge.

Variable	ED group (*N* = 80)	CI group (*N* = 80)	Total (*N* = 160)	*P* value
Postoperative time to discharge, *n* (%)				<.0001
(12 h,24 h]	42 (52.50)	-	42 (26.25)	-
(24 h,36 h]	11 (13.75)	-	11 (6.88)	-
(36 h,48 h]	27 (33.75)	-	27 (16.88)	-
(48 h,72 h]	-	33 (41.25)	33 (20.63)	-
>72h	-	47 (58.75)	47 (29.38)	-
Length of hospital stay, *n* (%)				<.0001
Discharged after 1 night	53 (66.25)	-	53 (33.13)	-
Discharged after 2 nights	27 (33.75)	-	27 (16.88)	-
Discharged after ≥3 nights	-	80 (100.00)	80(50.00)	-

### Subgroup

#### Age

A subgroup analysis stratified by age, with a cutoff of 65 years, was performed to evaluate AF recurrence. Among patients aged 65 years or older, 46 underwent early discharge and 40 underwent conventional inpatient hospitalization. No significant difference in 12-month AF recurrence was observed between the two groups (4.35% vs. 7.50%, *P* = 0.660). Kaplan–Meier analysis also showed comparable freedom from AF recurrence between the two groups in this subgroup, with no statistically significant difference (log-rank *P* = 0.419). Among patients younger than 65 years, 34 underwent early discharge and 40 underwent conventional inpatient hospitalization. Similarly, no significant 12-month difference in AF recurrence was found between the two groups (14.71% vs. 12.50%, *P* = 1.000), and Kaplan–Meier curves demonstrated no significant between-group difference in AF recurrence (log-rank *P* = 0.971). Details are presented in Figure 4.

**Figure 4:**
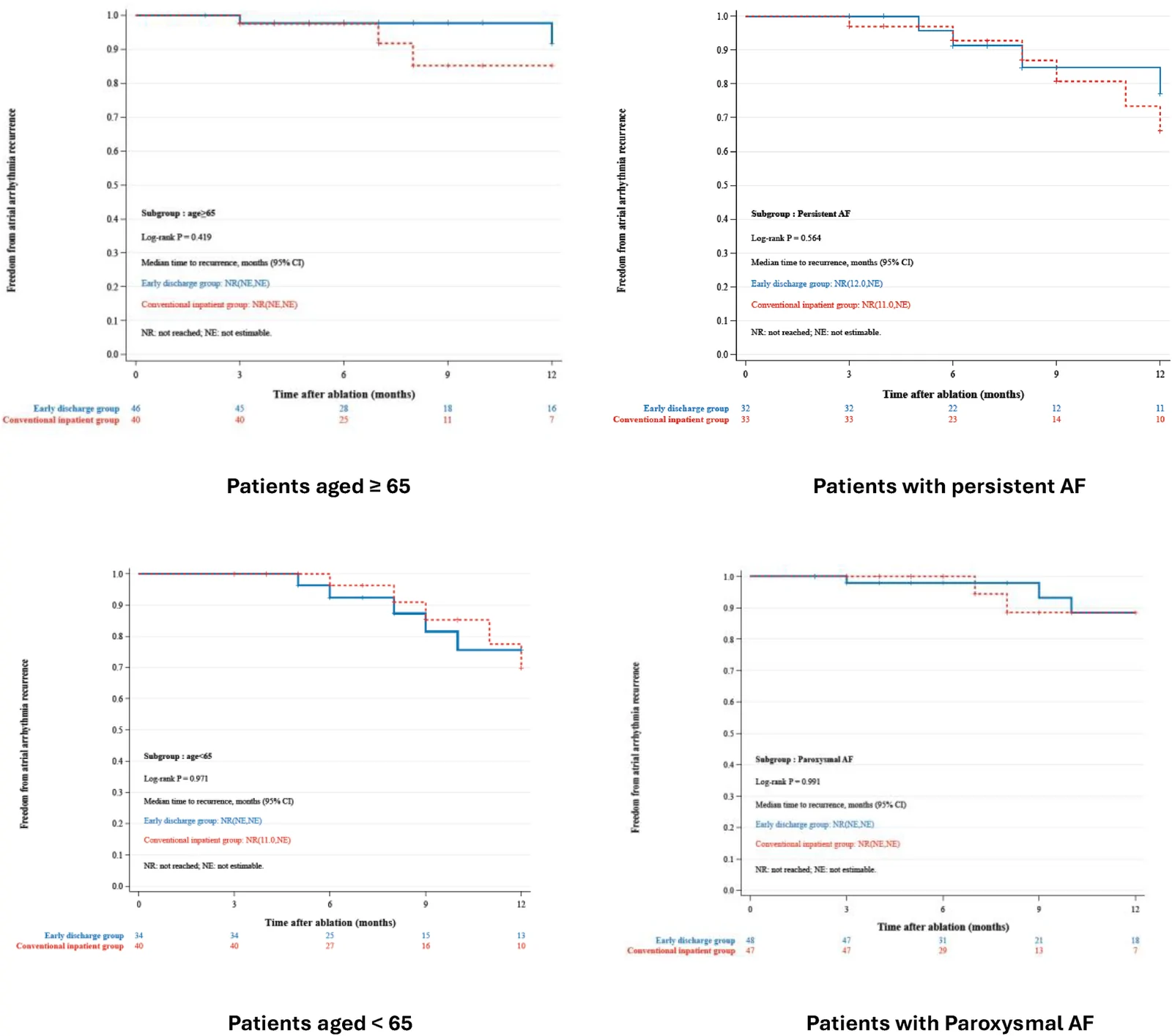
Kaplan–meier curves for AF recurrence in the subgroup analysis.

#### Atrial fibrillation type

A subgroup analysis stratified by atrial fibrillation type, including persistent and paroxysmal AF, was also performed. Among patients with persistent AF, 32 underwent early discharge and 33 underwent conventional inpatient hospitalization. No significant difference in 12-month AF recurrence was observed between the two groups (12.50% vs. 18.18%, *P* = 0.733). Kaplan–Meier analysis likewise showed no significant between-group difference in freedom from AF in this subgroup (log-rank *P* = 0.564). Among patients with paroxysmal AF, 48 underwent early discharge and 47 underwent conventional inpatient hospitalization. Similarly, no significant 12-month difference in AF recurrence was found between the two groups (6.25 vs. 4.26%, *P* = 1.000), and Kaplan–Meier curves demonstrated comparable freedom from AF recurrence (log-rank *P* = 0.991). Details are presented in Figure 4.

#### Economic analysis

There was no significant difference in total hospitalization costs between the ED and CI groups (RMB 83,986.15 ± 26,082.88 vs. RMB 84,804.33 ± 27,305.40, *P* = 0.957). However, the ED group incurred significantly lower medical service costs than the CI group (RMB 956.37 ± 345.81 vs. RMB 1,189.50 ± 534.90, *P* = 0.0003). Medication costs were also significantly lower in the ED group (RMB 1,558.69 ± 352.68 vs. RMB 1,684.95 ± 413.61, *P* = 0.035). No significant between-group differences were observed in diagnostic costs (*P* = 0.696), treatment costs (*P* = 0.826), or consumables costs (*P* = 0.795). Detailed hospitalization expenditures are presented in Table 4.

**Table 4:** Hospitalization cost comparison between the c group and inpatient groups.

Cost Category (RMB)	ED group (*n* = 80)	CI group (*n* = 80)	*P* value
Medical service cost	956.37 (345.81)	1,189.5 (534.9)	0.0003^a^
Diagnostic cost	6,028.72 (816.19)	6,150.37 (1,254.19)	0.6960^a^
Treatment cost	8,998.52 (2,684.83)	8,956.08 (2,299.05)	0.8262^a^
Medication cost	1,558.69 (352.68)	1,684.95 (413.61)	0.0354^a^
Consumables cost	66,443.84 (23,379.09)	66,802.89 (23,946.75)	0.7954^a^
Total hospitalization cost	83,986.15 (26,082.88)	84,804.33 (27,305.4)	0.9,565^a^

a Stands the Kruskal–Wallis test.

## Discussion

In this real-world study conducted at a high-volume cryoballoon ablation center in China, the early discharge pathway was associated with clinical outcomes comparable to those of conventional inpatient care. No significant difference was observed in atrial fibrillation recurrence during follow-up, and post-procedural complications were uncommon in both groups. In addition, the early discharge pathway was associated with lower medical service and medication costs, although total hospitalization costs were comparable between groups. Exploratory subgroup analyses further suggested that older age and persistent atrial fibrillation did not necessarily preclude selected patients from early discharge management. Collectively, these findings suggest that a structured early discharge pathway may be associated with improved healthcare resource utilization and acceptable in-hospital clinical outcomes in appropriately selected patients.

To our knowledge, this is the first clinical and economic evaluation of cryoballoon ablation for atrial fibrillation in a real-world early discharge setting in China. The result showed that the efficacy of ED group is comparable to CI group in the real-world setting. ED group had a slightly lower percentage of AF recurrence compared to CI group at 12 months (8.75% % vs. 10.00%), but without significant difference (*P* = 0.786). This finding suggests that management through an early discharge pathway did not compromise the effectiveness of cryoballoon ablation. Similar findings have been reported in several studies assessing same-day discharge after AF ablation. A recent systematic review and meta-analysis including 19 studies and 26,335 patients found that structured same-day discharge was not associated with increased mortality, major complications, readmissions, or emergency department visits compared with overnight hospitalization (18). Creta et al. reported low rates of early complications and readmissions in a large multicenter cohort of same-day discharge patients (19), while Deyell et al. further confirmed favorable outcomes across different ablation modalities including cryoballoon ablation and radiofrequency ablation (9). Similar findings have also been reported specifically for same-day discharge after cryoballoon ablation (20). However, these studies were conducted in Western same-day discharge programs, which are not directly comparable with the Chinese early discharge model. In Western healthcare systems, same-day discharge is often implemented within ambulatory surgery centers, where patients are discharged on the day of the procedure. By contrast, in China, day surgery generally refers to an early discharge pathway and is usually implemented in tertiary public hospitals **(**14). This difference is partly attributable to the concentration of healthcare resources in large hospitals, relatively limited primary-care support, and the ongoing development of reimbursement policies for day surgery in China (14, 21, 22). Consequently, evidence generated from Western same-day discharge programs may not be directly generalizable to the Chinese healthcare setting.

The overall safety profile was similar between the ED and CI groups. There was only one case of pericardial effusion in CI group within 48 h after procedure and it was treated urgently by the physician during the procedure, requiring a longer length of hospital stay. No complication was recorded in the ED group during the early post-procedural observation period. This finding is consistent with previous studies demonstrating the favorable safety profile of cryoballoon ablation. In a large multicenter US cohort of 2,374 patients undergoing cryoballoon ablation, immediate post-procedural complications were uncommon, with hematoma being the most frequently reported event (20), while Bartoletti et al. similarly observed only one transient phrenic nerve palsy among 143 selected patients managed with same-day discharge (23). What is more, large registry data suggest that cryoballoon ablation is associated with a low rate (4‰) of severe periprocedural complications (24). However, given the relatively small sample size and the lack of systematic post-discharge surveillance, the absence of major in-hospital complications should be interpreted cautiously, and the present study cannot definitively establish comparative safety between the two pathways.

An important exploratory finding of this study is that advanced age did not appear to substantially modify the observed association between discharge pathway and recurrence outcomes. No statistically significant difference in AF recurrence was observed between the ED and CI groups among patients aged ≥65 years (4.35% vs. 7.50%, *P* = 0.660). This finding is clinically relevant in China, where the AF population is ageing and clinicians may be cautious about applying shortened hospitalization pathways to older patients. Whether older age itself should influence eligibility for an early discharge strategy remains uncertain. In a recent real-world study of 301 octogenarians undergoing AF ablation, Wahedi et al. reported acceptable arrhythmia-free survival and low rates of major procedural complications (25). These findings suggest that chronological age alone may be insufficient to determine eligibility for an early discharge pathway following cryoballoon ablation. Nevertheless, our findings as an exploratory analysis were not powered to definitively assess the impact of age in early discharge. The current evidence base remains limited, particularly in elderly populations. Future prospective studies are warranted to identify which clinical characteristics, beyond age itself, are most relevant for selecting appropriate candidates for early discharge AF ablation.

In patients with persistent AF, no significant difference in AF recurrence was observed between the ED and CI groups (12.50% vs. 18.18%, p = 0.733). Persistent AF is generally associated with more advanced atrial structural and electrical remodeling and has been consistently linked to less favorable rhythm outcomes after catheter ablation compared with paroxysmal AF (26, 27). Therefore, clinicians may be concerned that an accelerated discharge pathway could adversely affect outcomes in this population. Our findings did not suggest an apparent association between early discharge management and increased recurrence risk among patients with persistent AF. However, these results should be interpreted cautiously because of the relatively small sample size and limited statistical power of this subgroup analysis. Further prospective studies with larger cohorts are needed to confirm whether early discharge management is associated with comparable outcomes among patients with persistent AF.

Despite a marked reduction in time to discharge and length of stay, the early discharge pathway did not result in a significant reduction in total hospitalization costs (RMB 83,986.15 ± 26,082.88 vs. RMB 84,804.33 ± 27,305.40, *P* = 0.957). This may be explained by the fact that treatment-related and consumable costs, which accounted for most overall expenditures, were comparable between groups. In contrast, significantly lower medical service costs (RMB 956.37 ± 345.81 vs. RMB 1,189.50 ± 534.90, *P* = 0.0003) and medication costs (RMB 1,558.69 ± 352.68 vs. RMB 1,684.95 ± 413.61, *P* = 0.035) were observed in the ED group, suggesting reduced use of hospitalization-related resources. These findings suggest that the main advantage of the early discharge pathway may lie in more efficient use of clinical resources and improved inpatient resource allocation, rather than in reducing the total direct cost of catheter ablation.

AF imposes a substantial economic burden worldwide, and catheter ablation, despite its higher upfront procedural costs, may provide long-term economic benefits by reducing future healthcare utilization (28). Accordingly, increasing attention has been directed toward strategies that further improve the efficiency of AF ablation care, including early discharge pathways (20, 29, 30). Kowalski et al. demonstrated that the duration of hospital stay was the primary driver of cost differences between same-day discharge and overnight-stay strategies after cryoballoon ablation, with per-patient savings ranging from US$917 to US$1,676 across different regions of the United States (20). He et al. likewise reported that an overnight stay following AF ablation was associated with approximately US$500 in additional hospital costs in the United Kingdom (30). Although these estimates cannot be directly extrapolated to China because of differences in healthcare systems and reimbursement structures, they consistently suggest that reducing post-procedural length of stay may improve healthcare resource utilization and may generate cost savings in selected healthcare settings.

Importantly, the potential value of early discharge pathways may extend beyond cost reduction alone. The 2024 EHRA/HRS/APHRS/LAHRS expert consensus statement recognizes same-day discharge as a reasonable strategy in appropriately selected patients undergoing AF ablation (7). In contrast, no specific recommendations regarding discharge timing currently exist in China, where prolonged post-procedural hospitalization remains common. Given the rapidly increasing prevalence of AF, the growing demand for catheter ablation, and persistent limitations in inpatient bed capacity at high-volume centers, early discharge pathways may provide an opportunity to improve patient throughput and optimize resource allocation without compromising clinical outcomes. Therefore, from both economic and healthcare system perspectives, implementation of a structured early discharge strategy may represent an efficient approach to AF care in appropriately selected patients.

### Study limitations

Several limitations of this study should be acknowledged. First, this was a retrospective, non-randomized, single-center study, and residual confounding cannot be completely excluded despite the use of propensity score matching analysis. Second, the reliance on intermittent ECGs may underestimate asymptomatic recurrences, which reflect real-world practice. Third, safety assessment was limited to in-hospital events, with post-discharge complications, emergency visits, and readmissions not systematically captured; therefore, delayed adverse events may have been missed, and the study was underpowered to compare safety outcomes between groups. Finally, the subgroup analyses according to age and AF type should be considered exploratory because of the relatively limited sample size and event rates. Larger multicenter prospective studies are needed to further evaluate the safety, effectiveness, and economic impact of early discharge pathways for AF ablation in China.

## Conclusion

In summary, our real-world analysis suggests that a structured early discharge pathway for cryoballoon ablation was not associated with a higher AF recurrence rate compared with conventional inpatient care, while reducing certain hospitalization costs and improving resource utilization in China. These findings support the feasibility of early discharge management in selected patients within experienced centers and suggest that this model may help address the growing demand for atrial fibrillation ablation. However, several limitations should be considered, including the retrospective single-center design, the potential for residual confounding despite propensity score matching, and the lack of systematic post-discharge surveillance for all safety events. In addition, the relatively limited sample size may restrict the generalizability of the findings. Therefore, larger prospective multicenter studies with comprehensive follow-up are needed to further validate the safety, effectiveness, and economic implications of this care model.

## Data Availability

The raw data supporting the conclusions of this article will be made available by the authors, without undue reservation.
