# Supplementary material for: Safety, effectiveness and healthcare resource implications of an early discharge pathway for cryoballoon ablation: real-world evidence from China
Source: Front Cardiovasc Med. 2026 Oct 26;13:1969824. doi: 10.3389/fcvm.2026.1969824 (PMC13745271; doi:10.3389/fcvm.2026.1969824)
Supplement: Supplementary file 1 [file Supplementaryfile1.pdf]

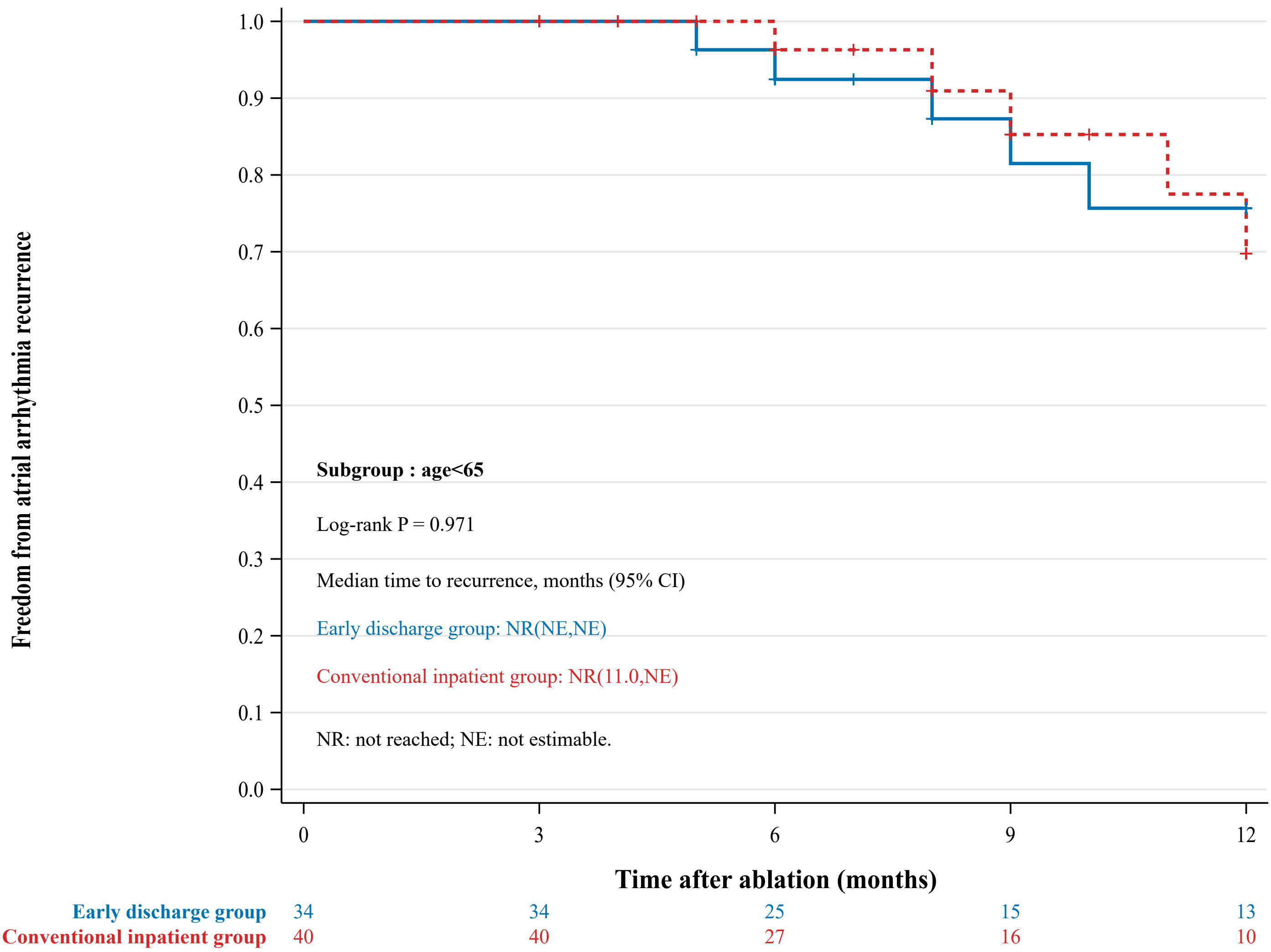
1.0
0.9
0.8
0.7
0.6
0.5
0.4
0.3
0.2
0.1
0.0
Freedom from atrial arrhythmia recurrence
Subgroup : age<65
Log-rank P = 0.971
Median time to recurrence, months (95% CI)
Early discharge group: NR(NE,NE)
Conventional inpatient group: NR(11.0,NE)
NR: not reached; NE: not estimable.
0
3
6
9
12
Time after ablation (months)
Early discharge group 34 34 25 15 13
Conventional inpatient group 40 40 27 16 10
