# Supplementary material for: Safety, effectiveness and healthcare resource implications of an early discharge pathway for cryoballoon ablation: real-world evidence from China
Source: Front Cardiovasc Med. 2026 Oct 26;13:1969824. doi: 10.3389/fcvm.2026.1969824 (PMC13745271; doi:10.3389/fcvm.2026.1969824)
Supplement: Supplementary file 2 [file Supplementaryfile2.pdf]

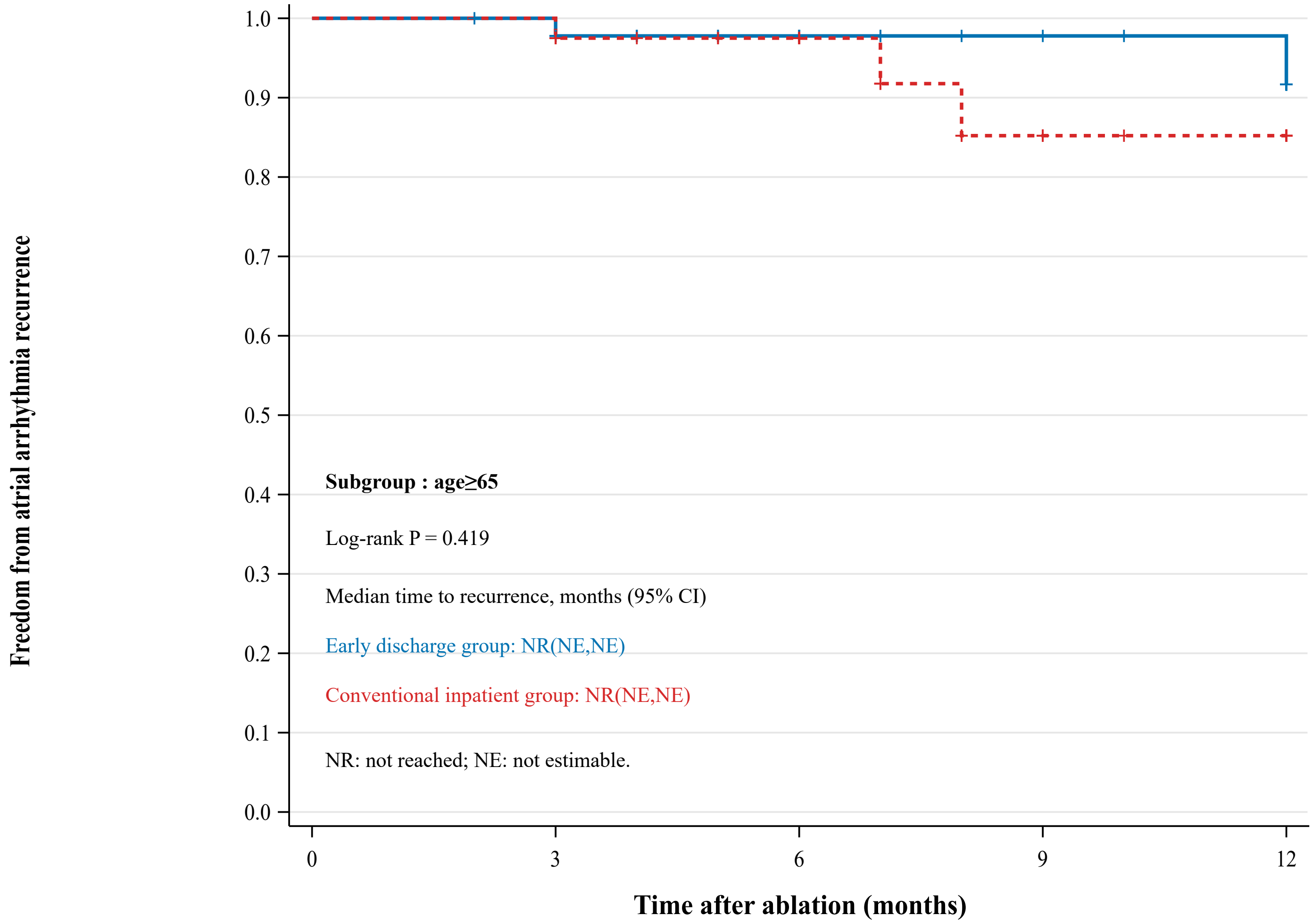

	0	3	6	9	12
**Early discharge group**	46	45	28	18	16
**Conventional inpatient group**	40	40	25	11	7
